# Supplementary material for: Genetic Variation of Physicochemical Properties and Digestibility of Foxtail Millet (Setaria italica) Landraces of Taiwan
Source: Molecules. 2019 Nov 26;24(23):4323. doi: 10.3390/molecules24234323 (PMC6930489; doi:10.3390/molecules24234323)
Supplement: Supplementary file 1 [file molecules-24-04323-s001.pdf]

S1 Table. The list of foxtail millet accessions studied by PI number, origin, and the apparent amylose content (AAC).

Accession	PI number	Origin [a]	Coordinates [b]	AAC (%)
383	PI433383	TTDARES, Taitung	121E09' 22N45'	1.57
385	PI433385	TTDARES, Taitung	121E09’ 22N45'	11.70
387	PI433387	TDAIS, Changhua	120E32' 24N00'	14.35
390	PI433390	Wutai, Pingtung	120E43' 22N45'	1.62
391	PI433391	Wutai, Pingtung	120E43' 22N45'	3.20
392	PI433392	Wutai, Pingtung	120E43' 22N45'	2.56
393	PI433393	Wutai, Pingtung	120E43' 22N45'	1.08
394	PI433394	Wutai, Pingtung	120E43' 22N45'	1.01
395	PI433395	Wutai, Pingtung	120E43' 22N45'	1.06
396	PI433396	Wutai, Pingtung	120E43' 22N45'	1.10
397	PI433397	Wutai, Pingtung	120E43' 22N45'	15.83
398	PI433398	Wutai, Pingtung	120E43' 22N45'	5.42
399	PI433399	Shinyi, Nantou	120E51' 23N42'	9.16
400	PI433400	Shinyi, Nantou	120E51' 23N42'	7.98
401	PI433401	Shinyi, Nantou	120E51' 23N42'	8.44
402	PI433402	Shinyi, Nantou	120E51' 23N42'	6.60
406	PI433406	Shinyi, Nantou	120E51' 23N42'	9.15
408	PI433408	Shinyi, Nantou	120E51' 23N42'	7.80
412	PI433412	Shinyi, Nantou	120E51' 23N42'	6.38
413	PI433413	Shinyi, Nantou	120E51' 23N42'	1.17
415	PI433415	Shinyi, Nantou	120E51' 23N42'	2.45
416	PI433416	Shinyi, Nantou	120E51' 23N42'	1.85
417	PI433417	Shinyi, Nantou	120E51' 23N42'	15.82
419	PI433419	Shinyi, Nantou	120E51' 23N42'	15.26
420	PI433420	Shinyi, Nantou	120E51' 23N42'	11.06
421	PI433421	Shinyi, Nantou	120E51' 23N42'	8.48
422	PI433422	Shinyi, Nantou	120E51' 23N42'	1.47
424	PI433424	Shinyi, Nantou	120E51' 23N42'	8.36
425	PI433425	Shinyi, Nantou	120E51' 23N42'	1.80
426	PI433426	Shinyi, Nantou	120E51' 23N42'	8.44
427	PI433427	Shinyi, Nantou	120E51' 23N42'	9.64

428	PI433428	Shinyi, Nantou	120E51' 23N42'	1.05
429	PI433429	Taimali, Taitung	121E00' 22N37'	1.10
430	PI433430	Taimali, Taitung	121E00' 22N37'	10.25
431	PI433431	Taimali, Taitung	121E00' 22N37'	10.31
432	PI433432	Taimali, Taitung	121E00' 22N37'	9.05
433	PI433433	Taimali, Taitung	121E00' 22N37'	0.80
434	PI433434	Taimali, Taitung	121E00' 22N37'	1.51
435	PI433435	Taimali, Taitung	121E00' 22N37'	1.76
436	PI433436	Taimali, Taitung	121E00' 22N37'	1.35
437	PI433437	Taimali, Taitung	121E00' 22N37'	1.65
438	PI433438	Daren, Taitung	121E53' 22N27'	1.74
439	PI433439	Daren, Taitung	121E53' 22N27'	1.23
440	PI433440	Daren, Taitung	121E53' 22N27'	0.69
441	PI433441	Daren, Taitung	121E53' 22N27'	1.78
445	PI433445	Lanyu, Taitung	121E33' 22N25'	7.78
448	PI433448	Haiduan, Taitung	121E10' 23N06'	7.94
449	PI433449	Haiduan, Taitung	121E10' 23N06'	2.32
451	PI433451	Haiduan, Taitung	121E10' 23N06'	6.96
455	PI433455	Haiduan, Taitung	121E10' 23N06'	10.22
456	PI433456	Haiduan, Taitung	121E10' 23N06'	7.45
458	PI433458	Beinan, Taitung	121E07' 22N47'	1.94
459	PI433459	Beinan, Taitung	121E07' 22N47'	1.36
460	PI433460	Beinan, Taitung	121E07' 22N47'	2.58
461	PI433461	Wutai, Pingtung	120E43' 22N45'	1.70
462	PI433462	Wutai, Pingtung	120E43' 22N45'	1.26
463	PI433463	Wutai, Pingtung	120E43' 22N45'	8.11
464	PI433464	Renai, Nantou	121E07' 24N01'	2.03
465	PI433465	Renai, Nantou	121E07' 24N01'	3.66
466	PI433466	Renai, Nantou	121E07' 24N01'	2.60
467	PI433467	Renai, Nantou	121E07' 24N01'	16.92
468	PI433468	Renai, Nantou	121E07' 24N01'	16.82
469	PI433469	Haiduan, Taitung	121E10' 23N06'	10.97
470	PI433470	Haiduan, Taitung	121E10' 23N06'	10.06
472	PI433472	Haiduan, Taitung	121E10' 23N06'	5.80
473	PI433473	Haiduan, Taitung	121E10' 23N06'	10.77
475	PI433475	Haiduan, Taitung	121E10' 23N06'	1.26
477	PI433477	Haiduan, Taitung	121E10' 23N06'	8.65
478	PI433478	Haiduan, Taitung	121E10' 23N06'	5.97

480	PI433480	Haiduan, Taitung	121E10' 23N06'	3.22
481	PI433481	Haiduan, Taitung	121E10' 23N06'	8.66
488		N. A.	N. A.	11.25
A261		SPNP, Taichung	121E03' 24N28'	8.24
A280		N. A.	N. A.	10.95
DL-1		Laiyi, Pingtung	120E39' 22N31'	2.16
DL-2		Laiyi, Pingtung	120E39' 22N31'	1.16
DNA-1		Beinan, Taitung	121E07' 22N47'	1.39
DNI-1		Dawu, Taitung	121E54' 22N21'	5.48
HY-2		Sandimen, Pingtung	120E39' 22N43'	1.30
HY-3		Sandimen, Pingtung	120E39' 22N43'	2.05
HY-4		Sandimen, Pingtung	120E39' 22N43'	1.86
LC-1		Beinan, Taitung	121E07' 22N47'	1.39
LC-2		Beinan, Taitung	121E07' 22N47'	7.46
LC-3		Beinan, Taitung	121E07' 22N47'	1.87
LC-5		Beinan, Taitung	121E07' 22N47'	2.04
ML-1		Maolin, Kaohsiung	120E40' 22N54'	15.20
ML-2		Maolin, Kaohsiung	120E40' 22N54'	14.11
SMCS-1		Jianshih, Hsinchu	121E12' 24N43'	1.56
SMCS-2		Jianshih, Hsinchu	121E12' 24N43'	1.78
TTS-5		TTDARES, Taitung	121E09' 22N45'	2.98
TU-1		Taitung	121E06' 22N73'	1.79
WT-1		Wutai, Pingtung	120E43' 22N45'	1.15

[a]TTDARES: Taitung District Agricultural Research and Extension Station, COA; TDAIS: Taichung District Agricultural Research and Extension Station, COA; SPNP: Shei-Pa National Park; N. A. indicates the origin of the accession was not recorded.

[b]The coordinates are based on the location of the townships because the foxtail millet townships were dated where the seeds were collected from or requested from the locals. N. A. indicates the origin of the accession was not recorded.

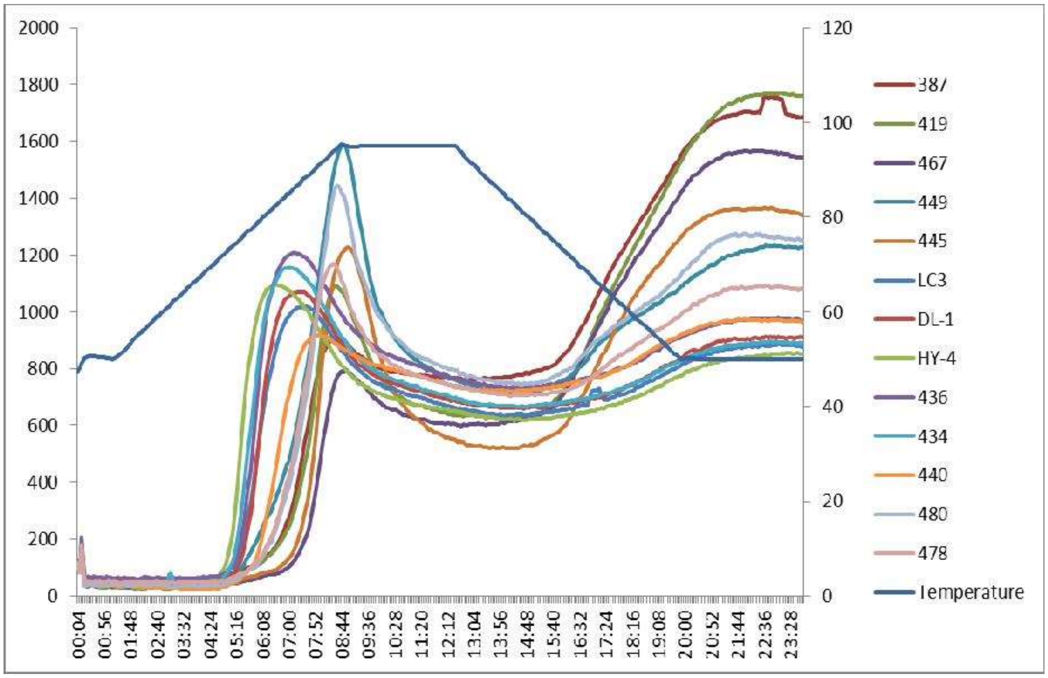

Figure S1. The RVA profiles of the selected 13 foxtail millet accession

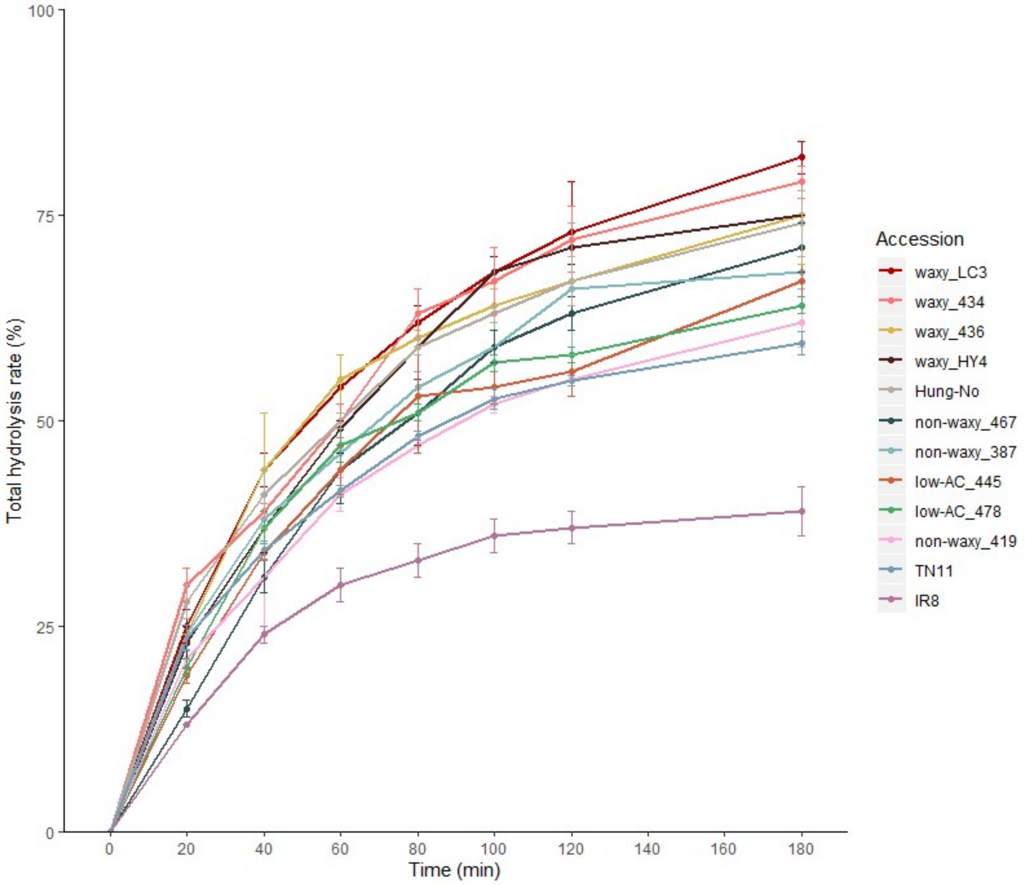

Figure S2. Starch hydrolysis curve of 9 foxtail millet accessions and 3 rice cultivars.
